# The impact of COVID-19 on women’s reproductive system

**DOI:** 10.3389/fmed.2024.1485022

**Published:** 2024-11-20

**Authors:** Shixiang Dong, Xia Liu, Yankui Wang

**Affiliations:** ^1^Department of Obstetrics and Gynecology, The Affiliated Hospital of Qingdao University, Shinan, China; ^2^Department of Reproductive Medicine, The Affiliated Hospital of Qingdao University, Qingdao, China

**Keywords:** reproductive system, menstruation, ovary, COVID-19, pregnant women

## Abstract

The coronavirus disease 2019 (COVID-19) pandemic has been a major global focus since 2019. However, drug development and vaccination have been unable to stop the rise in the number of COVID-19 infections. As a result, almost everyone has been infected with COVID-19. As the COVID-19 pandemic nears its end, it is important to explore whether contracting COVID-19 has any irreversible effects on the female reproductive system. This article aims to review the effects of COVID-19 on the female ovary and reproductive system and examine whether these effects are permanent. In conclusion, we can state that COVID-19 has not caused any long-lasting effects on the reproductive health of most women, with the exception of a few cases where premature ovarian failure has been observed. These temporary effects, such as menstrual disturbances and temporary fertility loss, tend to diminish and eventually disappear over time.

## Introduction

This year marks the fourth year of the COVID-19 pandemic. Due to vaccinations and the continuous mutations of the novel coronavirus, the pathogenicity has decreased along with the proportion of severely ill patients. However, due to the strong infectivity of the new mutant strains, the number of confirmed patients has not decreased significantly. Nowadays, almost everyone has been infected with COVID-19. It has been reported that during the COVID-19 pandemic, the virus can attack male testicles, which can lead to a decrease in male fertility (1). Whether COVID-19 has an impact on the female reproductive system and whether these effects are reversible have attracted people’s attention. Could COVID-19 infection lead to a decline in female fertility? Does it affect a woman’s ovarian function? How does COVID-19 affect women’s menstruation? What is the impact of COVID-19 infection on pregnant women? This review attempts to find answers to these questions and discuss the impact of COVID-19 on the female reproductive system from various aspects and whether these effects are reversible.

## Results

### COVID-19 can cause ovarian dysfunction without affecting ovarian reserve

Some studies have found that organs with high angiotensin-converting enzyme 2 (ACE2) expression may be attacked by the COVID-19 virus (2–4). Some studies have reported that male reproductive endocrine function may be impaired due to COVID-19 infection. Similarly, ovaries may also be a target for COVID-19 due to high ACE2 expression. However, there is no clear clinical evidence showing the effects of COVID-19 on ovarian function. Although some have reported COVID-19 patients developing premature ovarian failure, these reports are still focused on individual cases, and there is no clear evidence that COVID-19 can cause premature ovarian failure (5, 6).

Li et al. found that mean sex hormone and anti-mullerian hormone (AMH) concentrations in women of childbearing age infected with COVID-19 did not differ from age-matched controls (7). Hence, the impact of COVID-19 infection on ovarian reserve might be minimal. However, Herrero et al. (8) found that novel coronavirus infection may damage ovarian function by changing the follicular microenvironment, leading to ovarian dysfunction and potentially affecting reproductive outcomes. But, in this study, patients were only analyzed for 3–9 months after novel coronavirus infection. They were not followed up for a longer period of time to determine whether these ovarian changes could be recovered after a longer period of time. However, Bentov et al. (9) showed no measurable differences in the steroidogenic mechanism of follicles and their hormonal environment after infection with severe acute respiratory syndrome coronavirus 2 (SARS-COV-2). They noted that COVID-19 did not have any measurable harmful effects on follicular function. Therefore, although COVID-19 changes the microenvironment of follicles leading to ovarian dysfunction, it does not have meaningful changes in indicators of ovarian reserve function such as AMH.

### COVID-19 infection briefly caused changes in women’s menstruation

It is well known that there are many forms of viruses that can affect the female reproductive endocrine system (10, 11). The emphasis on the effects of COVID-19 on female sex hormones, menstruation, and fertility increased due to the worldwide COVID-19 pandemic in 2019. Li et al. conducted the first study on how COVID-19 affects women’s menstruation. Out of the 177 patients they examined, 25% experienced alterations in their menstrual volume and 28% experienced changes in their menstrual cycle. These changes were mainly due to decreased menstrual volume (20%) and prolonged menstrual cycle (19%) (7). According to Li et al., these menstrual changes could be attributed to temporary disruptions in sex hormone levels as a result of the suppression of ovarian function, which can be restored quickly within a short span of time (7). Similarly, Madendag et al. (12) observed that alterations in menstrual status could be associated with intense immune reactions and inflammation, along with psychological stress and anxiety due to COVID-19 sickness. These scholars all pointed out that changes in menstrual status due to COVID-19 are not permanent and will resolve within a few months after the infection has cleared.

### COVID-19 may have some impact on women’s fertility due to the existence of ACE2 receptors

As sperm is vulnerable to viral infection (13), COVID-19 infection affects male testicles to the extent of affecting male fertility (1, 14). Similarly, some studies have shown that COVID-19 may affect female fertility and interfere with female reproductive function (15, 16). There exists both direct and indirect proof indicating that COVID-19 could potentially harm female fertility (17). In addition, research has indicated that pregnant women with mild COVID-19 infection might carry the virus in the placenta, resulting in complications such as fetal growth restriction and other issues related to pregnancy (18). At present, currently, evidence suggests that COVID-19 may disrupt female reproductive function, causing menstrual irregularities, infertility, and fetal distress (15).

ACE2 is a receptor for COVID-19 (19); therefore, all organs expressing ACE2 are likely to be affected by COVID-19. It is important to mention that ACE2 exhibits high expression in the ovaries and has a significant role in controlling reproduction (20). In addition, ACE2 plays a role in regulating follicular development, ovulation, angiogenesis, and the degeneration of the corpus luteum. Further, it regulates changes in endometrial tissue and embryonic development (15). Consequently, COVID-19 might affect a woman’s fertility by targeting ovarian tissue and granulosa cells or by damaging endometrial epithelial cells.

### Estrogen has some protective effects against COVID-19

Epidemiological evidence shows that there are gender differences in the severity and mortality of COVID-19. The severity and prognosis of COVID-19 in pre-menopausal women are less than that of males of the same age (21–24). Nearly twice as many men experience severe symptoms or die from COVID-19 compared to women (25, 26). Similar results were found in an Italian study, where the authors suggested that estrogen could reduce disease progression and promote viral clearance by acting on the immune system (27–29). Whereas in Africa, despite predictions by public health experts to have the worst outcomes during the COVID-19 pandemic, morbidity, hospitalization, and mortality rates have been lower compared to other continents. Dufailu et al. (30) attributed this to higher estrogen levels in women of African descent, and speculated that estrogen may offer some protection against novel coronavirus infection. In a meta-analysis conducted in 2020, it was confirmed that males were at greater risk for COVID-19 disease (31). This protective effect of estrogen is believed to be caused by the regulation of estrogen, especially E2, on inflammatory response and immune cell function. Estrogen not only has immunomodulatory effects but also plays a role in regulating the expression of Th1 and Th2 cytokines. It inhibits excessive inflammatory processes, and restores homeostasis, which may inhibit the cytokine storm syndrome in women (a condition thought to be a major cause of COVID-19 morbidity and mortality) (32–34). In addition, experimental data indicate that estrogen may have a direct antiviral impact on SARS-CoV-2 through the suppression of ACE-2 mRNA expression in bronchial epithelial cells.

In addition, Liu et al. reported that because estrogen levels in women declined after menopause, estrogen no longer provided beneficial effects, and there was no difference in male and female morbidity in patients over 55 years of age (35). Loss of ovarian function during menopause and the resulting changes in sex hormone concentrations may increase the risk of COVID-19 infection (36).

For most infectious diseases, women have consistently been observed to have a stronger immune response than men. The immune system of women, generally speaking, exhibits a greater ability to respond to pathogens by producing more interferons and antibodies. Nevertheless, postmenopausal women experience a reduction in this protective effect, primarily due to the decreased influence of estrogen (37). Some studies have shown that estradiol treatment can significantly reduce the risk of death in postmenopausal women (38). The protective effect of estrogen against COVID-19 was also supported by a higher survival rate among women with COVID-19 who received hormone replacement therapy (HRT) in a cohort study (39). There seems to be a general consensus that estrogen therapy can effectively combat COVID-19. According to Chanana et al. hormonal treatment for COVID-19 patients can be short-term without long-term side effects due to their reversible effects (40).

### The impact of COVID-19 on mothers and newborns

COVID-19 infection in newborns is another major concern. No health complications were reported in 33 newborns born to mothers with COVID-19, except for four cases of shortness of breath, according to a study conducted in Wuhan, China (41). Other studies, with a smaller sample size, did not document any health issues in newborns except for low birth weight (<2,500 g) and premature birth (42, 43). Overall, infants born to mothers with COVID-19 do not seem to be more susceptible to clinical complications compared to typical pregnancies, and certain observed problems in newborns might be linked to the mother’s overall well-being rather than stemming directly from the COVID-19 infection.

There is evidence suggesting that ACE2 receptors are present in the placenta and uterus, thereby elevating the potential for SARS-CoV-2 transmission from the mother to the fetus (44). However, the hypothesis is not supported by most published data, as the majority of newborns to mothers infected by COVID-19 test negative (45–47). Although, COVID-19 can be detected in breast milk, suggesting that there is an opportunity for transmission of COVID-19 through breastfeeding. Transmission during breastfeeding is still a possibility, even if the breast milk does not contain any viruses. Given the weakened immune system of newborns, it is recommended that babies of peripartal patients confirmed with COVID-19 be artificially fed or begin breastfeeding 14 days after recovery and discharge from hospital. We fully endorse the implementation of all essential measures to prevent the spread of the virus to infants, such as practicing hand hygiene prior to handling infants and wearing facial masks while breastfeeding (48). We encourage mothers who plan to breastfeed to utilize a separate breast pump, and it is crucial to adequately sterilize the pump after each use to greatly reduce the risk of infection in infants and young children.

Vaccination appears to be the most effective way to prevent the adverse consequences of SARS-CoV-2 infection on mothers and their fetuses (49). However, vaccination rates among pregnant women are significantly lower than in the general population of the same age. It was not until evidence supporting the safety and effectiveness of COVID-19 vaccination during pregnancy emerged that rates of COVID-19 vaccination among pregnant and breastfeeding women began to increase (49).

Assisted reproductive technology is crucial for infertile couples desiring to become parents, and delaying treatment can significantly decrease their chances of success (50). However, the COVID-19 pandemic has had a profound impact on medically assisted reproductive procedures. As the pandemic nears its end and its effects on assisted reproduction lessen, we must reflect on this experience, ready ourselves for future pandemics, and safeguard the rights of infertile couples to fulfill their dream of becoming parents.

## Discussion

Over the time, with the continuous mutation of COVID-19, the virulence is declining. The death rate of COVID-19 is also decreasing due to the emergence of COVID-19 vaccines and drugs. However, due to the immune escape mechanism of the new variant strain, the infectivity of COVID-19 is stronger than that of the original strain. As a result, the number of people diagnosed with COVID-19 has not decreased significantly, and nowadays, almost everyone has been infected with COVID-19 at some point.

This review discusses various aspects of the impact of COVID-19 on women’s reproductive system. We have discussed and made recommendations for pregnant women infected with COVID-19. In some women, changes in menstruation caused by COVID-19 infection may be the result of transient hormonal changes caused by suppressed ovarian function, and these changes will quickly return to normal as the patient recovers. Therefore, there is no need to worry about menstrual disorders after COVID-19 infection. COVID-19 infection may damage ovarian function by changing the follicular microenvironment, leading to ovarian dysfunction and potentially affecting reproductive outcome. However, this conclusion also requires long-term follow-up and clinical evaluation to verify whether the ovarian function of COVID-19 patients returns to normal. Some studies have shown that COVID-19 does not affect ovarian reserves. Although, COVID-19 may have had an impact on female fertility due to the widespread presence of ACE2 receptors in the reproductive system. Estrogen may also offer some protection to women infected with COVID-19, allowing pre-menopausal women with COVID-19 to have less severe disease and a better prognosis than men of the same age. For newborns of mothers infected with COVID-19, there does not appear to be an increased risk of clinical complications compared to normal pregnancies. However, due to the weak immune system of newborns, we recommend that mothers infected with COVID-19 take extra care when feeding their newborns to avoid infection as much as possible.
